# Live-attenuated Japanese encephalitis virus inhibits glioblastoma growth and elicits potent antitumor immunity

**DOI:** 10.3389/fimmu.2023.982180

**Published:** 2023-04-11

**Authors:** Zhongbing Qi, Jing Zhao, Yuhua Li, Bin Zhang, Shichuan Hu, Yanwei Chen, Jinhu Ma, Yongheng Shu, Yunmeng Wang, Ping Cheng

**Affiliations:** ^1^ State Key Laboratory of Biotherapy and Cancer Center/Collaborative Innovation Center for Biotherapy, West China Hospital, Sichuan University, Chengdu, China; ^2^ Department of Biotherapy, Cancer Center, West China Hospital of Sichuan University, Chengdu, China; ^3^ Department of Arboviruses Vaccine, National Institute for Food and Drug Control, Beijing, China

**Keywords:** glioblastoma, oncolytic virotherapy, anti-tumor immunity, aPD-L1, live-attenuated Japanese encephalitis vaccine virus, oncolytic virus

## Abstract

Glioblastomas (GBMs) are highly aggressive brain tumors that have developed resistance to currently available conventional therapies, including surgery, radiation, and systemic chemotherapy. In this study, we investigated the safety of a live attenuated Japanese encephalitis vaccine strain (JEV-LAV) virus as an oncolytic virus for intracerebral injection in mice. We infected different GBM cell lines with JEV-LAV to investigate whether it had growth inhibitory effects on GBM cell lines in vitro. We used two models for evaluating the effect of JEV-LAV on GBM growth in mice. We investigated the antitumor immune mechanism of JEV-LAV through flow cytometry and immunohistochemistry. We explored the possibility of combining JEV-LAV with PD-L1 blocking therapy. This work suggested that JEV-LAV had oncolytic activity against GBM tumor cells in vitro and inhibited their growth in vivo. Mechanistically, JEV-LAV increased CD8+ T cell infiltration into tumor tissues and remodeled the immunosuppressive GBM microenvironment that is non-conducive to immunotherapy. Consequently, the results of combining JEV-LAV with immune checkpoint inhibitors indicated that JEV-LAV therapy improved the response of aPD-L1 blockade therapy against GBM. The safety of intracerebrally injected JEV-LAV in animals further supported the clinical use of JEV-LAV for GBM treatment.

## 1 Introduction

Glioblastoma (GBM) is the most common and aggressive form of primary brain tumor. The current standard care for GBM involves the removal of the main tumor part through surgery, followed by radiotherapy and adjuvant therapy with systemic chemotherapeutic drugs (1). Although GBM patients respond to these multiple approaches; their response is mediocre and the median survival is <24 months (2). Immunotherapy is effective against other tumors; however, the clinical trials of immunotherapy for GBM have not provided satisfactory results (3–5).

Oncolytic viruses (OVs) can selectively kill infected tumor cells and effectively induce antitumor immunity. The recombinant oncolytic poliovirus PVSRIPO recently showed potential in a phase I study involving recurrent GBM patients by extending the overall median survival period of treated GBM patients to 2 years (6).

Japanese encephalitis virus (JEV) is a single-stranded positive-sense RNA flavivirus transmitted by mosquitoes. It is closely related to other emerging viral pathogens, including dengue fever virus, yellow fever virus, West Nile virus, and zika virus (ZIKV). Like most neurotropic flaviviruses, after entering the brain, JEV can infect pericytes, astrocytes, and microglia, preferentially targeting developing neurons and neuron precursors (7). These characteristics of flaviviruses allow the use of OVs for GBM treatment. For instance, preclinical studies have demonstrated that ZIKV can eliminate GBM in the mouse model by targeting GBM stem cells (GSCs) and CD8^+^ T cell-mediated antitumor immunity (8, 9). Therefore, we explored whether JEV can be used as an OV for GBM treatment.

The live attenuated Japanese encephalitis vaccine strain (JEV-LAV) SA14-14-2 has been administered to more than 300 million children since its clinical approval in China and other countries in 1989. Further, no vaccine-related encephalitis cases have yet been reported from these countries (10). No reports of JEV-LAV being used as an OV are currently available. Therefore, we here explored the use of JEV-LAV SA14-14-2 as an OV and elucidated its underlying action mechanism in GBM treatment.

## 2 Materials and methods

### 2.1 Animals and cell lines

Six-week-old C57BL/6J female mice were purchased from Beijing Huafukang Bioscience (Beijing, China). 4T1, A549, 3T3, BHK21, and GBM cell lines, including GL261, A172, T98G, U87, and U251, were purchased from ATCC and cultured in DMEM (Hyclone) media supplemented with 10% FBS (HyClone) at 37°C in the presence of 5% humidified CO_2_. Then, GL261 cells with a luciferase reporter gene (*GL261-luc*) were established *via* viral transduction and puromycin selection (2 µg/mL). All cell lines were negative for mycoplasma.

### 2.2 JEV strains

The JEV-LAV used in the present study was an attenuated JEV SA14-14-2 strain, which was kindly gifted by the Arbovirus Vaccine Laboratory of China Institutes for Food and Drug Control. The vaccine virus strain was screened as described previously (10). Briefly, the vaccine virus strain SA14-14-2 was derived from a wild-type JEV SA14 isolated from a pool of Culex pipiens mosquito larvae. The SA14 virus was attenuated through a continuous passage in primary hamster kidney (PHK) cell cultures. After 100 passages of the virus in PHK cells, followed by thrice cloning of plaque, one less virulent clone 12-1-7 was selected from 36 plaque clones. The selected virus was amplified in Vero cells and purified by ultrafiltration, and the viral titers were quantified with BHK21 cells purchased from ATCC.

### 2.3 TCID50

BHK-21 cells (1 × 10^4^) were seeded into 96-well plates and incubated overnight in 100 μL of DMEM medium supplemented with 2% FBS. The vaccine strain SA14-14-2 was serially diluted 10-fold in 100 μL of DMEM medium supplemented with 2%FBS and transferred into 96-well plates. Then, an equal volume of DMEM medium supplemented with 2% FBS was added into the two columns that served as control. After 10 days of incubation, the plates were read to confirm the endpoint (cytopathic effect). The viral titers were calculated according to the method described by Reed and Muench. Three repeated plates were used to measure the titers.

### 2.4 *In vitro* viral infection and cell proliferation assay

GL261 cells were plated in 12-well plates and infected with JEV-LAV at an MOI of 0, 1, 5, and 25. The cells were stained with crystal violet staining solution (Sigma) for 5 min after 48 h of the infection.

GL261 cells were plated in 96-well plates and infected with JEV-LAV at an MOI of 0, 1, 5, and 25. The cell survival rates were calculated by using the Cell Counting Kit-8 (MedChem Express) after 48 h of the infection.

GL261, A172, T98G, U87, U251, 4T1, 3T3, and A549 cells were plated in 96-well plates and infected with JEV-LAV at an MOI 25. Cytotoxicity was evaluated by using CCK8 after 48 h of the infection.

GL261, U87, 4T1, and A549 cells were infected with JEV-LAV at an MOI of 10 for 72 h, and the resultant supernatant was collected. The viral titers in the cell supernatant were quantified by TCID50 with BHK21 cells.

### 2.5 Tumor implantation and treatment

GL261-luc cells (1 × 10^5^) infected with 1 × 10^6^ PFU JEV-LAV for 2 h were implanted into the right frontal lobe of mice by using stereotactic tumor establishment apparatus. A suspension of the same number of GL261-luc cells and inactivated JEV-LAV (achieved by heating in a 56°C water bath for 30 min) were injected as a control. After 5 days of implantation, we monitored the tumor development in mice through bioluminescence imaging.

Using the same coordinates as mentioned earlier, we implanted the single-cell suspension of GL261-luc cells (1 × 10^5^ cells in 10 μL) into the right frontal lobe of the experimental mice. After 5 days of implantation, we randomly selected the mice with confirmed tumor formation and injected them intratumorally with 1.6 × 10^6^ PFU JEV-LAV. The injection of inactivated JEV-LAV served as a control. On days 6, 8, 12, and 14 after implantation, the mice received an intraperitoneal injection of αPD-L1 antibodies (200 μg) or PBS.

GL261 cells (1 × 10^6^ cells suspended in 100 µL of phosphate-buffered solution) were injected into the right flank. After 12 days of implantation, we randomly grouped the mice with confirmed tumor formation and injected them intratumorally with 1.6 × 10^6^ PFU JEV-LAV. The injection of inactivated JEV-LAV served as a control. On days 13, 15, 17, and 19 after implantation, the mice received an intraperitoneal injection of αPD-L1 antibodies (200 μg) or PBS. All animal experiments were performed as per the guidelines of the Animal Care and Use Committee of West China Hospital, Sichuan University, China.

### 2.6 Bioluminescence imaging

The tumor development in the animals was monitored through bioluminescence imaging. After receiving the intraperitoneal injection of D-fluorescein (150 mg/kg, Gold Bio), the animals were anesthetized with isoflurane (2%) and imaged with the IVS50 imaging system (PerkinElmer). The data were analyzed with the Living Image 2.6 software (PerkinElmer).

### 2.7 Hematoxylin and eosin, immunohistochemical, and immunofluorescence staining

When the tumor-bearing mice in the control group displayed classic neurological symptoms, such as curling up into lumps, the two groups of mice were perfused with normal saline and 4% paraformaldehyde solution, and their brains were isolated. The brain tissues were then fixed in 4% paraformaldehyde solution for 48 h (SigmaAldrich), embedded in paraffin, and sliced into 5-μm-thick sections. These paraffin sections were deparaffinized in xylene (twice, 5 min each), gradually hydrated in a gradient series of alcohol (100%, 90%, and 70%, for 5 min each), and stained with hematoxylin and eosin (SigmaAldrich). To analyze the tumor-infiltrating immune cells, we sequentially incubated the treated sections with the primary antibodies (CD3, Abcam) and secondary antibodies, followed by staining of the nuclei with hematoxylin. To analyze the replication of the virus in a tumor, these brain sections were incubated with a primary mouse anti-JEV NS3 protein antibody (JeneTex; 1:500 dilution) overnight at 4°C and goat anti-mouse IgG conjugated with TRITC (1:500) at 37°C for 60 min, after which the nuclei were stained with DAPI (1:1,000 dilution) for 10 min at room temperature. The relevant images were acquired by confocal laser scanning microscopy (Zeiss).

### 2.8 Virus RNA detection by quantitative real-time PCR

The experimental mice were treated with inactivated JEV-LAV and JEV-LAV as described earlier and sacrificed on day 5 of the treatment, after which their brain and tumor tissues were harvested and weighed. Viral RNA was extracted with the Viral RNA Extraction Kit (Hengya). The viral RNA was quantified by qRT-qPCR using the Japanese Encephalitis Virus Detection Kit (Xybio) as per the manufacturer’s instruction.

### 2.9 Flow cytometry analysis

To analyze the effect of JEV-LAV on infiltrating immune cells in tumors and the PD-L1 expression of GBM tumor cells, we sacrificed the tumor-bearing mice 17 days after tumor inoculation (12 days after the administration). The brain-tumor quadrants were excised from the sacrificed mice and digested into a single-cell suspension. For the detection of the PD-L1 expression in GBM tumor cells *in vivo*, we only stripped the tumor tissues. After filtering through a 70-μm filter, the dead cells were stained with the Fixable Viability Stain 620 (BD Biosciences). The single-cell suspension was then incubated with Fc-block (BD Biosciences) for 10 min before staining. The following antibodies were used based on the cell surface staining procedure: APC/cy7 anti-CD3, FITC anti-CD4, PE/cy7 anti-CD8, PE anti-PD-1, APC anti-TIM-3, PE anti-CD25, APC/cy7 anti-CD45, Percp anti- CD11b, FITC anti-F4/80, APC anti-Gr-1, and PE anti-CD206. PE anti-Foxp3 was stained intracellularly according to the intracellular-staining protocol.

### 2.10 Safety evaluation of intracerebral administration of JEV-LAV in mice

Using the same methods as mentioned earlier, 6-week-old C57BL/6J female mice were intracerebrally inoculated with 1.6 × 10^6^ PFU (consistent with the therapeutic dose) JEV-LAV, while PBS injection served as control. After injection, we monitored the physical state and weight of the mice every week for 2 weeks. Two weeks after the injection, we conducted various tests, including open field, elevated plus maze, rota-rod, and tail suspension, as described previously (8).

### 2.11 Statistical analyses

All data were analyzed using GraphPad Prism8. The statistical significance of total photon flux from the tumors was calculated by Sidak’s multiple-comparisons test. Kaplan–Meier survival curves were employed to assess the animal survival, and statistical significance was analyzed by the log-rank test. The statistical significance of other experiments was analyzed by unpaired Student’s *t*-test. *P < 0.05; **P < 0.01; ***p < 0.0001; ns, no statistical significance.

### 2.12 Study approval

The animal experiments were approved by the West China Hospital of Sichuan University Biomedical Ethics Committee, and all experiments conformed to all relevant regulatory standards.

## 3 Results

### 3.1 Safety evaluation of intracerebrally administered JEV-LAV

**Figure 1:**
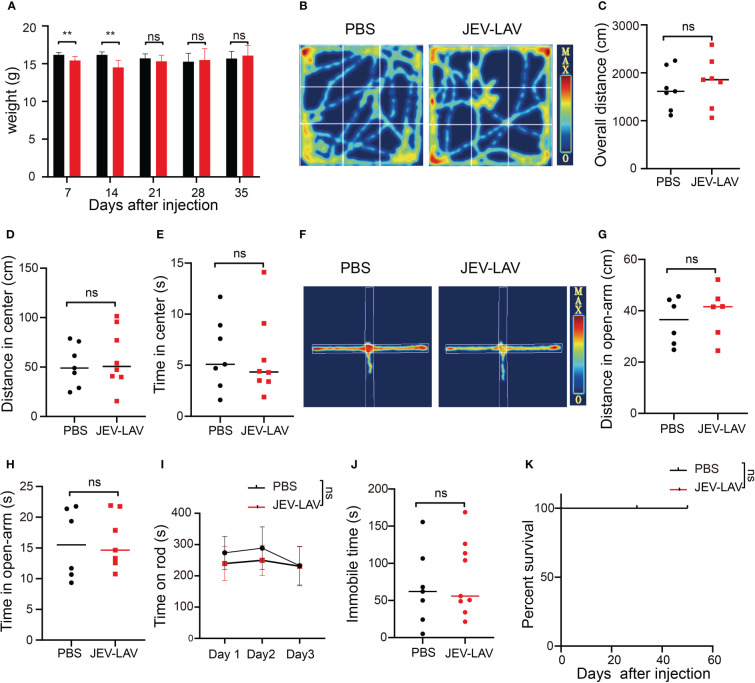
Safety evaluation of intracerebral administration of JEV-LAV in the experimental mice. **(A)**Weight changes in the mice after 2 weeks of injection. **(B–J)** C57BL/6J mice were intracerebrally inoculated with 1.6 × 10^6^ PFU (consistent with the therapeutic dose) JEV-LAV, while PBS injection served as control. The clinical signs and survival of the animals were monitored daily. Behavioral testing was conducted after 2 weeks. **(B)** The motion track of the mice in the open field test. **(C)** Overall distance covered by the mice in the open field test. **(D)** Movement distance in the center of the mice in the open field test. **(E)** Residence time in the center of the mice in the open field test. **(F)** The motion tracking of mice in the elevated plus-maze test. **(G)** The overall distance of mice in the open arm. **(H)** The residence time of mice in the open arm. **(I)** Total time of mice on the rod in the rota-rod teat. **(J)** Immobile time of mice within 5 min of tail suspension in the tail-suspension test. **(K)** Survival (n = 8/group). *P < 0.05; **P < 0.01; ***P < 0.0001; ns, no statistical significance.

To test the safety of JEV-LAV, four behavioral tests were conducted to determine whether intracerebrally administered JEV-LAV causes adverse reactions in mice. These tests were the open field test, elevated plus maze test, tail suspension test, and rotarod test. We injected 1.6 × 10^6^ PFU JEV-LAV intracranially into C57BL/6J mice. At 2 weeks after JEV-LAV administration, no abnormalities were observed in the mice (erected fur, diarrhea, loss of appetite, lethargy, and gait instability), except a slight weight loss (**Figure 1A**). The mice restored the lost weight in the third week. Results of the behavioral tests conducted after 2 weeks revealed that JEV-LAV- or PBS-injected mice exhibited the same degree of spontaneous exploration behavior, anxiety, depression, and exercise ability (**Figures 1B–J**). Until the 50th day of observation, no mouse from any of the groups died (**Figure 1K**). These results indicate that JEV-LAV is safe for intracerebral injection as OVs.

### 3.2 Oncolytic activity of JEV-LAV against GBM tumor cells *in vitro*

**Figure 2:**
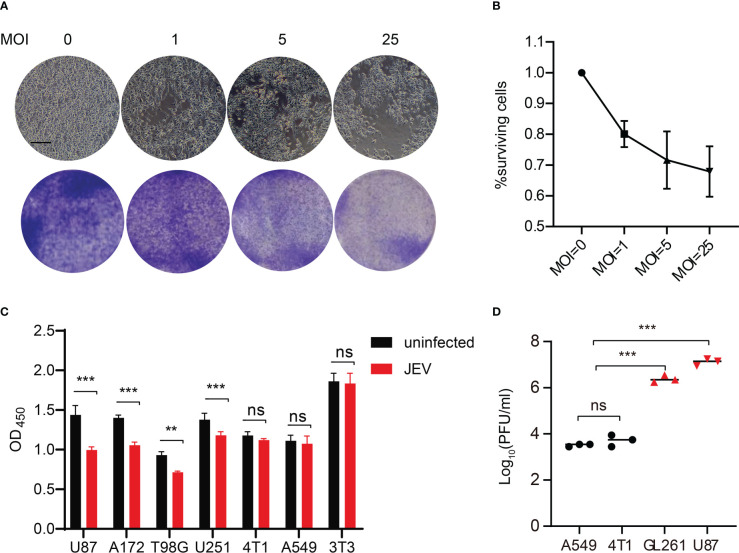
JEV-LAV specifically infects and kills GBM cells, but does not harm other non-nervous system tumor cells. **(A)** Representative images of microscopy and crystal violet staining of GL261 cells infected with JEV-LAV. Scale bars = 200 μm. **(B)** The inhibitory effect on mouse GBM GL261 cells infected at different MOI. The surviving rate of cells infected at an MOI of 0 was set to 100%. Data were pooled from three independent experiments. **(C)** Cell viability of human GBM cells (U87, A172, T98G, and U251), human lung cancer cells (A549), mouse breast cancer cells (4T1), and mouse embryonic fibroblast cell (NIH 3T3) after 48 h of JEV-LAV infection (MOI = 25). **(D)** The replication of JEV-LAV in GBM cells and other non-nervous system tumor cells. **P < 0.01; ***P < 0.001; ns, no statistical.

First, the inhibitory effect of JEV-LAV on GBM GL261 (mouse glioma 261) cells infected at different multiplicities of infection (MOI: 0, 1, 5, and 25) was investigated after 48 h (**Figures 2A, B**). JEV-LAV dose-dependently inhibited GL261 growth. Because no report demonstrates the role of JEV in GBM treatment, we continued to characterize the oncolytic effect of JEV-LAV on several GBM cell lines to investigate whether JEV-LAV exhibits a broad killing effect. Thus, human A172, T98G, U87, and U251 cells were infected, and JEV-LAV was found to significantly inhibit the growth of all the GBM cells after 48 h (**Figure 2C**). However, JEV-LAV exhibited no inhibitory effect against the growth of breast and lung cancer cells. Similarly, JEV-LAV had no inhibitory effect on the growth of the mouse embryonic fibroblast cell line (NIH 3T3) (**Figure 2C**). Furthermore, the JEV-LAV infectious titers in the culture supernatant of GBM cells were significantly higher than those in the culture supernatant of other non-nervous system tumor cells (**Figure 2D**).

### 3.3 JEV-LAV inhibits GBM growth *in vivo* and prolongs the survival of tumor-bearing mice

**Figure 3:**
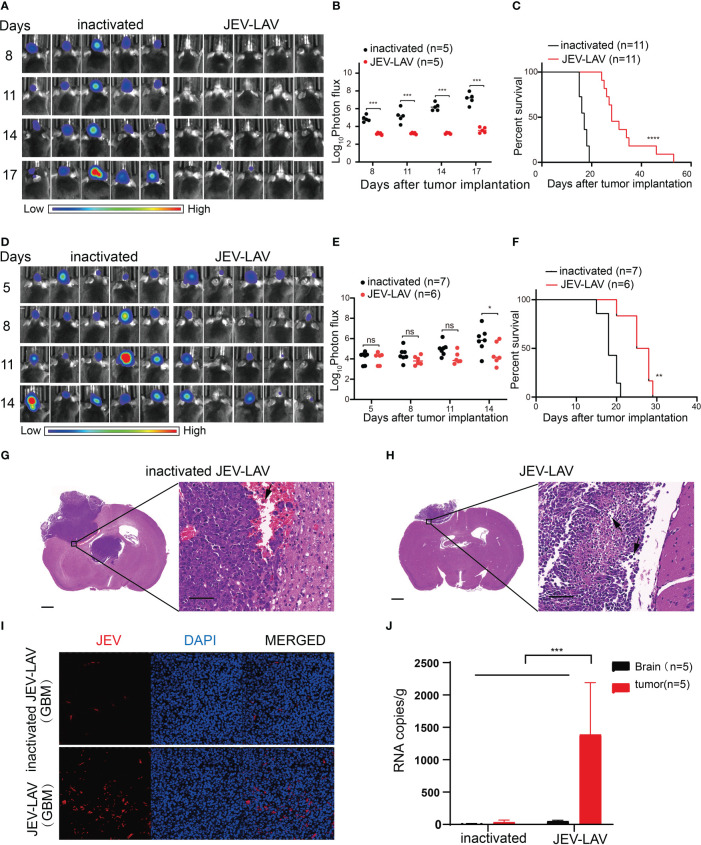
JEV-LAV inhibits the growth of GBM *in vivo* and prolongs the survival of tumor-bearing mice. **(A–C)** C57BL/6J mice were intracranially implanted with 1 × 10^5^ GL261-luc cells infected with 1 × 10^6^ PFU JEV-LAV for 2 h or HI-JEV-LAV (control). **(A)** Individual tumor growth *via* bioluminescence at the indicated times. **(B)** Quantification of total photon flux from each animal. **(C)** Survival. **(D–J)** C57BL/6J mice were intracranially implanted with 1 × 10^5^ GL261-luc cells and injected intratumorally with 1.6 × 10^6^ PFU JEV-LAV or HI-JEV-LAV after 5 days. **(D)** Individual tumor growth *via* bioluminescence at the indicated time points. **(E)** Quantification of total photon flux from each animal. **(F)** Survival. **(G, H)** Representative pictures of hematoxylin and eosin staining of coronal brain slices on day 12 after JEV-LAV treatment. Scale bars = 2000 μm (left panel) and 50 μm (right panel). The immune cells infiltrating into the tumor are indicated by arrows. **(I)** Representative pictures of immunofluorescent staining of the glioma paraffin sections for JEV NS3 protein (red) and DAPI (blue). **(J)** Distribution of JEV-LAV in the brain and tumor of mice after 5 days of intracerebral administration. *P<0.05, **P<0.01, ***P<0.001,****p<0.0001, ns no statistical significance.

We used two models for evaluating the effect of JEV-LAV on GBM growth in mice. For the *in vivo* tumor formation experiment, non-inactivated and inactivated JEV-LAV-infected GL261 cells were intracranially injected into immunocompetent mice. Bioluminescence imaging revealed rapid GBM progression in mice inoculated with inactivated JEV-LAV-infected GL261 cells; all mice exhibited classic neurological symptoms and died within 19 days (**Figures 3A–C**). However, the mice inoculated with non-inactivated JEV-LAV-infected GL261 cells exhibited slow GBM progression (**Figures 3A, B**), and the median survival time was significantly extended from 17 days to 28 days (**Figure 3C**).

To analyze the JEV-LAV therapeutic efficacy *in vivo*, we intracranially injected GL261-luc cells into the right hemisphere of mouse brains. After 5 days, we confirmed tumor formation through bioluminescence and intratumorally injected the tumor-bearing mice with non-inactivated or inactivated JEV-LAV. Bioluminescence imaging revealed that the signal intensities of GBM tumors in the non-inactivated JEV-LAV-injected tumor-bearing mice were significantly weaker than those in inactivated JEV-LAV-injected mice (**Figures 3D, E**). In addition, GBM progression was slower in the non-inactivated JEV-LAV-injected mice than in the inactivated JEV-LAV-injected mice. Moreover, the median survival time of the non-inactivated JEV-LAV-injected mice was 8 days more than that of the inactivated JEV-LAV-injected mice (**Figure 3F**). Hematoxylin and eosin staining of brain sections revealed that the tumor size was smaller in the non-inactivated JEV-LAV group than in the inactivated JEV-LAV group on the 17th day after tumor implantation (12 days after JEV-LAV treatment). Tumors in the non-activated JEV-LAV group mice had fewer bleeding points and slower tumor progression than those in the inactivated JEV-LAV group mice (**Figures 3G, H**). Immunofluorescence staining revealed that the virus antigen was abundantly expressed in tumors from the JEV-LAV-treated mice (**Figure 3I**). In addition, viral RNA accumulation in the brain and tumors of the treated mice suggested that JEV-LAV selectively replicates in the tumor tissues (**Figure 3J**).

### 3.4 JEV-LAV increases D8^+^ T cell infiltration into tumor tissues

**Figure 4:**
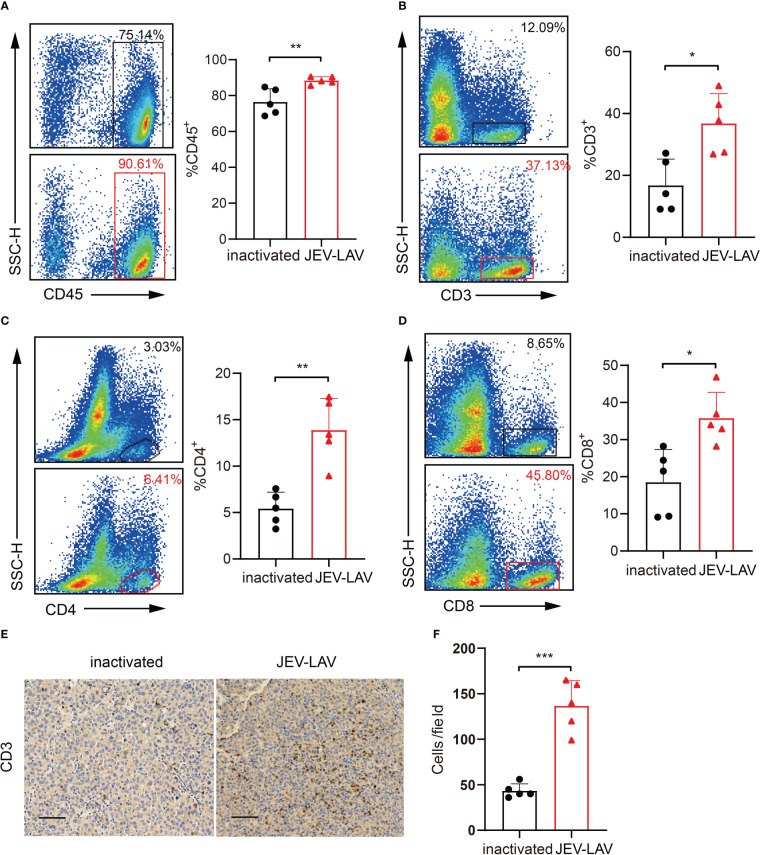
JEV-LAV treatment increases the infiltration of CD8^+^ T-cells into the tumor tissues. C57BL/6J mice were intracranially implanted with 1 × 10^5^ GL261-luc and injected intratumorally with 1.6 × 10^6^ PFU JEV-LAV or inactivated JEV-LAV after 5 days. On day 17 after tumor implantation, the tumors were harvested, dissociated, stained with fluorescent dye-conjugated anti-mouse antibodies, and analyzed by multicolor FACS. Representative flow cytometry scatter plots and statistical analyses of the percentage of **(A)** CD45^+^cells, **(B)** CD3^+^ cells, **(C)** CD4^+^ cells in CD3^+^ cells, and **(D)** CD8^+^ cells in CD3^+^ cells. **(E)** Representative images of CD3 immunohistochemical staining of the coronal brain sections at day 17 of tumor inoculation. Scale bars = 50 μm. **(F)** Quantification of the numbers of CD3^+^ cells in each field of view of the microscope at 20×. The cell counts were obtained from at least three random fields/tumor sections. n = 4 or 5 per group. *P<0.05, **P<0.01, ***P<0.001.

Using flow cytometry, we analyzed immune cells in tumor tissues 17 days after tumor implantation. The JEV-LAV group tissues had significantly increased (P < 0.05) number of CD45^+^ total leukocytes, CD3^+^ T cells, CD3^+^ CD4^+^T cells, and CD3^+^ CD8^+^T cells compared with the control group tissues (**Figures 4A–D**, **Figure S1A, B**). Consistent with the flow cytometry results, CD3 staining of the coronary brain slices also indicated that tumor infiltration with lymphocytes increased after JEV-LAV injection (**Figures 4E, F**). Overall, JEV increased lymphocyte infiltration in tumors, thereby improving the immune lymphocyte deficiency in GBM.

### 3.5 JEV-LAV remodels the immunosuppressive GBM microenvironment

**Figure 5:**
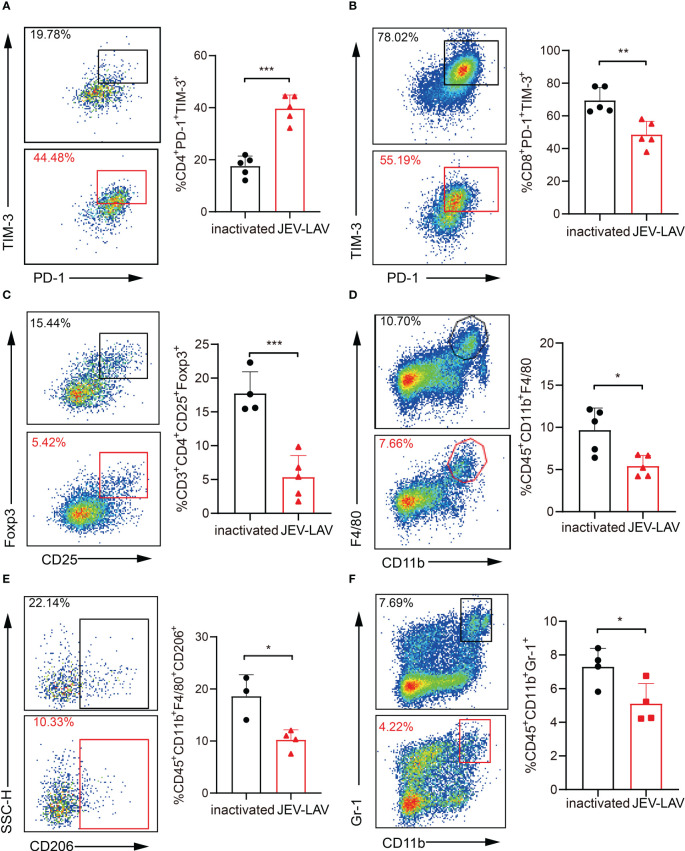
JEV-LAV remodels the immunosuppressive GBM microenvironment. The experimental mice were treated as described in **Figure 4**. The representative flow cytometry scatter plots and statistical analysis of the percentage of **(A)** PD-1^+^TIM-3^+^ cells in CD4^+^ cells, **(B)** PD-1^+^TIM-3^+^ cells in CD8^+^ cells, **(C)** CD25^+^Foxp3^+^ cells in CD4^+^ cells, **(D)** CD11b^+^F4/80^+^ cells in CD45^+^ cells, **(E)** CD206^+^ cells in CD11b^+^F4/80^+^ cells, and **(F)** CD11b^+^Gr-1^+^ cells in CD45^+^ cells. n = 4 or 5 per group. *P < 0.05; **P < 0.01; ***P < 0.001.

The major cellular players mediating GBM immunosuppression are CD4^+^ CD25^+^ Foxp3^+^ T regulatory cells (Tregs), myeloid-derived suppressor cells (MDSCs), and M2 polarized tumor-associated macrophages (TAMs). Moreover, the immune checkpoint coinhibitory receptors PD-1, CTLA-4, and TIM-3 highly expressed in T cells compete with the costimulatory receptor CD28 for binding to ligands CD80 and CD86, thereby inhibiting T cell activation. Therefore, we investigated whether JEV-LAV treatment can remodel the immunosuppressive GBM microenvironment. According to the flow cytometry analysis, although PD-1^+^TIM-3^+^ CD4^+^ T cell infiltration increased in the tumors of the JEV-LAV-injected mice (**Figure 5A**, **Figures S1B**, **S2AB**), this infiltration decreased significantly (**Figure 5B**, **Figures S1B**, **S2A, B**). Similarly, the number of CD4^+^CD25^+^ Foxp3^+^ Tregs and the ratio of Tregs to CD8+ T cells also significantly decreased (P < 0.05) (**Figure 5C**, **Figure S1C**). On analyzing the myeloid cells, we noted that the decrease in the number of M2-TAMs (CD45^+^CD11b^+^F4/80^+^CD206^+^) may have resulted in the decrease in the total number of TAMs (CD45^+^CD11b^+^F4/80^+^) (**Figure 5D, E**, **Figure S1A**). At the same time, MDSC (CD45^+^CD11b^+^Gr-1^+^) infiltration was significantly reduced (P < 0.05) (**Figure 5F**, **Figure S1A**). These observations indicate that JEV-LAV therapy can effectively transform the immunosuppressive GBM microenvironment into an immunostimulatory state conducive to immunotherapy.

### 3.6 JEV-LAV therapy improves the response of aPD-L1 blockade therapy

**Figure 6:**
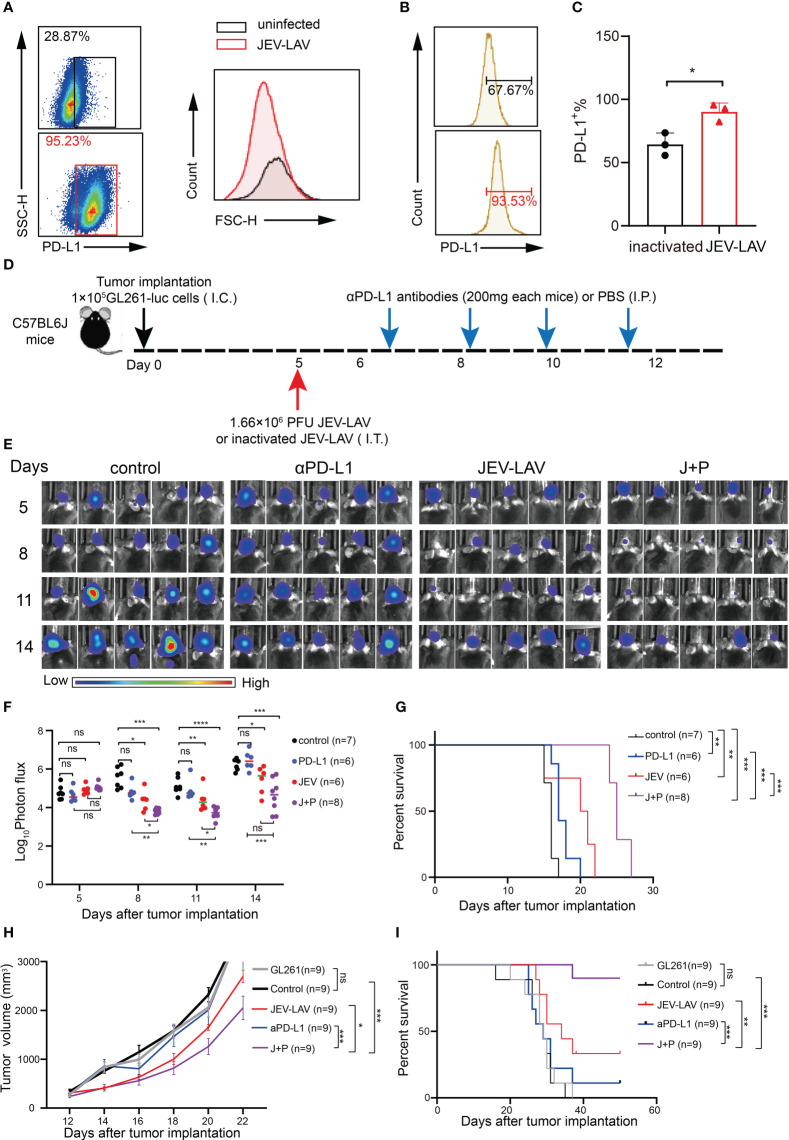
JEV-LAV therapy enhances the response of aPD-L1 blockade therapy. **(A)** The PD-L1 expression in GL261 cells infected with JEV-LAV *in vitro*. **(B)** PD-L1 expression in tumor cells *in vivo* after JEV-LAV treatment. **(C)** Percentage of PD-L1^+^ cells among the total cells isolated from the tumor. **(D)** Treatment schedule. C57BL/6J mice were intracranially implanted (I.C.) with 1 × 10^5^ GL261-luc cells, injected intratumorally (I.T.) with 1.6 × 10^6^ PFU JEV-LAV or inactivated JEV-LAV on day 5 and then injected intraperitoneally (I.P.) αPD-L1 antibodies (200 mg) on days 6, 8, 10, and 12. **(E)** Individual tumor growth *via* bioluminescence at the indicated time points. **(F)** Quantification of the total photon flux from each animal. **(G)** Survival. **(H)** Tumor volume. **(I)** Survival. *P<0.05, **P<0.01, ***P<0.001,****p<0.0001, ns no statistical significance.

Viral infection triggers the innate immune system-mediated IFN-γ response. IFN-γ upregulation stimulates tumor cells to increase the expression of immunomodulatory molecules (such as programmed cell death ligand 1, PD-L1). These molecules negatively regulate the antitumor immune response. Therefore, we tested whether *in vitro* infection and intratumoral injection of JEV-LAV change PD-L1 expression in GL261 cells and GBM tumor cells, respectively. PD-L1 expression was upregulated after 48 h of *in vitro* JEV-LAV infection (**Figure 6A**, **Figure S2C**). Similarly, the percentage of PD-L1^+^ cells among total GBM tumor cells 1 week after intratumoral JEV-LAV injection was close to 100% (**Figures 6B, C**).

We further evaluated whether JEV-LAV treatment in combination with anti-PD-L1 antibody (αPD-L1) can enhance the overall therapeutic effect in GL261 orthotopic glioma mice. JEV-LAV and αPD-L1 antibodies were injected intratumorally and intraperitoneally in the glioma mice, respectively, according to the schedule (**Figure 6D**). Bioluminescence imaging revealed that the signal intensities of GBM tumors in mice injected with JEV-LAV+α-PD-L1 were significantly weaker than those in the control group mice (P < 0.05), JEV-LAV-injected mice, and α-PD-L1-injected mice (**Figure 6E, F**). The median survival time of mice treated with JEV-LAV+α-PD-L1 was significantly prolonged compared with that of those in other groups (**Figure 6G**).

Typically, in GL261 studies, tumors are not detected on magnetic resonance imaging scans before days 10–14. Therefore, to investigate the efficacy of JEV-LAV+α-PD-L1 in the treatment of larger tumors, *in vivo* antitumor studies were conducted on C57/Bl6 mice bearing subcutaneous GL261 GBMs. The results of tumor volume and survival curve suggest that JEV-LAV enhances the antitumor effect of α-PD-L1 therapy (**Figures 6H, I**).

## 4 Discussion

GBM is among the most fatal malignant brain tumors. An in-depth understanding of GBM classification and underlying pathogenesis has been achieved through comprehensive molecular profiling technology; however, precision oncology approaches have not offered any satisfactory clinical insights (11, 12). This is mainly because of adaptive mutations and drug resistance resulting from molecular heterogeneity and redundant signaling pathways in GBM (13, 14). Cancer immunotherapy has recently made remarkable progress. The monoclonal antibodies ipilimumab, nivolumab, and pembrolizumab block the immune checkpoint and induce long-lasting remission of many cancer types. These antibodies have received approval for the treatment of various malignant tumors including melanoma, urothelial bladder cancer, head and neck squamous cell carcinoma, non-small cell lung cancer, and classical Hodgkin lymphoma (15, 16). Furthermore, cellular immunotherapy for cancers has achieved immense success in the treatment of hematological malignancies. *Tisagenlecleucel* and *axicabtagen-ciloleucel*, the two CD19-targeting chimeric antigen receptor T cell therapies, have been successfully applied for the treatment of acute lymphoblastic leukemia and diffuse large B-cell lymphoma. These therapies have also received FDA and EMA approval for clinical use (17, 18). Therefore, immune-based therapeutic approaches are good options for GBM.

The concept of the central nervous system (CNS) with immune privilege originates from the preliminary experimental data reported by Peter Medawar’s team 60 years ago (19, 20). Until 2015, the brain was believed to lack special lymphatic channels, which were speculated to limit antigen presentation from the brain to immune cells. However, recent data have expanded our understanding of active immune mechanisms in the CNS. CNS resident cells include microglia in the brain parenchyma (tissue-resident cells, TRM) and border-related macrophages (BAMs). TRMs support neuron development and function (21). BAMs are located in the CNS boundary area, which is related to their special barrier function and immune regulation (22). Moreover, the CNS in a stable state has been examined immunologically (23, 24). After infection in the CNS, the interaction between infiltrating T cells and activated microglia can maintain the function of T effector cells in CNS parenchyma (23, 25). Thus, these findings support that although the brain is a different part of immunology, the immune microenvironment provides sufficient opportunities for immunotherapy for the treatment of brain tumors.

However, immunotherapy for GBM has limited efficacy compared with that for other solid tumors. Several factors may limit the efficacy of immunotherapy against GBM. First, brain tumors with low lymphocyte infiltration are less responsive to immunotherapy (26). Second, the immunosuppressive GBM microenvironment is the primary reason for the aforementioned limited efficacy, especially for T cells, including immunosuppressive immune cells (Tregs, MDSCs, and TAMs), tumor cell-derived inhibitory cytokines, and the upregulated expression of immune checkpoint receptors on T cells and of PD-L1 on tumor cells. In addition to the immunosuppressive GBM microenvironment, a lot of evidence indicates that systemic GBM immunosuppression is also a major problem associated with conventional treatments and immunotherapy (27–29). Furthermore, the inability of ICIs to cross the blood–brain barrier (BBB) and disrupt immune checkpoint signaling *in situ* may be a reason for only a subset of advanced cancer patients responding to a single-agent immune checkpoint blockade (30). Some studies have shown that neurotropic viruses enter the CNS by breaching the BBB (31, 32). One study showed that laboratory-attenuated rabies virus, which is the same neurotropic virus as JEV, enhances BBB permeability (33).

OVs are natural or genetically modified viruses that specifically infect and lyse tumor cells, leading to the release of tumor antigens and induction of an antitumor immune response (34). Oncolytic virotherapy is a novel type of cancer immunotherapy. We here found that JEV-LAV has excellent replication ability and a potent oncolytic effect on GBM cells. JEV-LAV did not inhibit the growth of non-nervous system tumor cells and healthy cells. Furthermore, we conducted *in vivo* anti-mouse glioma experiments in immunocompetent mice to investigate the effect of JEV-LAV on the GBM immune microenvironment. Subsequently, the tumor tropism of JEV-LAV on human GBM cells will be examined in nude mice in the following experiments. The pre-existing attenuation properties of SA14-14-2 (the JEV-LAV used in this study) as a human vaccine strain are also crucial for being OV candidates (35). Currently, the oncolytic platforms for GBMs derived from human vaccine strains, including the vaccinia virus, poliovirus, and MVs, have entered the clinical stage (36). SA14-14-2 has cleared the clinically reliable safety verification by being used for vaccinating children and has been monitored for genetic stability and neurotoxicity through intracranial inoculation in mice (37–39). Although Chen et al.’s study demonstrated that JEV-LAV intracranially injected into 3-week-old BALB/c nude mice can induce lethal neurovirulence. Notably, intracranially injected JEV-LAV did not cause the death of C57BL/6J mice in our study. The neurovirulence of JEV-LAV has been tested in several laboratory animals, including weaned mice and rhesus monkeys (10). In immunocompetent mice, a dose of intracranially administered 10^6^ PFU of JEV-LAV was deemed non-lethal (40). However, in rare cases, mice died with intracranially injected JEV-LAV (41). This extremely low incidence is related to several factors influencing the experiment, such as mice age and strain, particularly the differences in the mouse genetic background (42). For example, a noticeable variability in mortality was reported when two lineages of age-matched outbred ICR mice were intracranially inoculated with a mutant of ChimeriVax-JE that contained two amino acid substitutions (F^107^L and K^138^E) in the SA14-14-2 E protein-coding region. Therefore, the difference between our and past study results may be attributable to differences in mouse strains. Thus, JEV-LAV is a safe candidate OV agent for GBM treatment at this stage.

The conflict between OV and host immunity has always been controversial. Pre-existing antiviral immunity generally leads to premature OV clearance and causes the damage to the systemic delivery, replication, and therapeutic gene expression of OVs. The relative contribution of immune responses against tumors and/or viruses in OV efficacy is being debated (43). A study demonstrated that the efficacy of oHSV in GBM treatment correlates with tumor- and viral antigen-specific cytotoxic T-cell infiltration (44). Furthermore, recent results have suggested that the antiviral response is an opportunity for GBM OV therapy. For example, a study showed that tumor-bearing mice immunized with newcastle disease virus (NDV) before treatment exhibited better therapeutic effects than naive mice (45). Coincidentally, by comparing the efficacy and immune response of immature mice or mice immunized with VG161, an HSV-1 OV in a phase 2 clinical trial, another study reported that pre-existing antiviral immunity may enhance OV-induced antitumor immunity (46). Studies have shown that anti-virus T cells may cross-react with tumor-associated antigens homologous to viral peptides and actively participate in tumor clearance (47). Many current methods attempt to prevent antibody-mediated virus neutralization, such as those involving liposomes, nanoparticles, and other OV delivery systems under development, and virus retargeting or chemical modification (48–51).

OVs can remodel the tumor microenvironment, which is the key reason for their antitumor effect. First, OVs universally induce a large T cell infiltration to transform “cold” tumors into “hot” tumors. Here, JEV-LAV also increased CD8^+^ T cell infiltration into the tumors. Second, OV can also reduce the number of mediate immunosuppression-mediating immune cells in the tumor microenvironment to offer favorable immune stimulation conditions for T-cell activation. For instance, oncolytic Adv targeted GBM by reducing Treg infiltration and increasing the number of IFNγ^+^CD8^+^T cells (52). Furthermore, in NDV-treated tumors, IFN-γ^+^ T cell infiltration increased, whereas MDSC accumulation decreased (53). Our results also revealed that JEV-LAV reduced the number of Tregs and MDSCs in tumor tissues. JEV-LAV treatment also reduced the total number of TAMs, possibly due to a significant reduction in M2-TAMs. Treatment of a subcutaneous xenograft tumor model in nude mice with oncolytic VV GLV-1H68 significantly upregulated the expression of pro-inflammatory cytokines (IFN-γ, IP-10, and M-CSF-1) and enhanced pro-inflammatory macrophage infiltration (54).

Although OVs are considered a promising cancer treatment strategy, the ideal antitumor effect cannot be achieved with OV monotherapy. OV combination therapy represents the ideal therapeutic strategy for future exploration. The GBM microenvironment creates a greater challenge to single oncolytic virotherapy. Innate immune cells and GSCs inhibit the effective replication and spread of viruses. GBM tumor cells exhibit a high degree of immunosuppression, high PD-L1 expression on tumor cells and its upregulation on TAMs, and a high expression of immune checkpoints on T cells. Therefore, OV-induced CD8^+^ T cells infiltrating tumor tissues may not have sufficient activity. Nowadays, GBM treatment strategies involving ICIs are under continuous development (55–57). Several clinical trials testing ICIs for GBM are underway, including ipilimumab (blocks CTLA-4) and nivolumab (blocks PD-1) (NCT04817254, NCT03576612). The OV+ICI combination therapy is worthy of further investigation. On the one hand, OVs can increase the effectiveness of ICIs in GBM with low T-cell infiltration by recruiting T cells and inducing an antitumor T-cell response. On the other hand, OV-induced upregulation of PD-1 expression on T cells and PD-L1 on tumor cells after an inflammatory response also increases GBM sensitivity to ICIs (58, 59). Through *in vitro* and *in vivo* experiments, we showed that after JEV-LAV infection, the expression of the immune escape molecule PD-L1 increases, providing basic support for the combined PD-1/PD-L1 blockade. Subsequent *in vivo* antitumor experiments also revealed an increased therapeutic effect of this combination compared with OV monotherapy.

The current results highlighted the great potential of JEV-LAV as an OV for GBM treatment. We also demonstrated the excellent therapeutic effect of JEV-LAV on glioma-bearing mice. JEV-LAV-induced remodeling of the GBM microenvironment was further used for immune checkpoint-blocking therapy. Moreover, this study presented an excellent safety profile of JEV-LAV, further backing its use as cerebral oncolytic virotherapy.

## Data availability statement

The original contributions presented in the study are included in the article/**Supplementary Material**. Further inquiries can be directed to the corresponding author.

## Ethics statement

The animal study was reviewed and approved by West China Hospital of Sichuan University.

## Author contributions

ZQ designed the research, performed the experiments, and wrote the initial manuscript. JZ participated in the revision of the manuscript and completed relevant experiments. YL provided the Live-attenuated Japanese encephalitis vaccine virus strain. PC provided constructive guidance to the experiment design and contributed to the final manuscript. ZQ, BZ, and SH were involved in the construction of the GBM orthotopic animal models and treatments. SH and other authors were involved in the flow cytometry assay. All authors read and approved the final manuscript.

## Supplementary material

Supplementary Figure 1 Gating strategies for all flow experiments. **(A)** Gating strategies for CD45^+^cells, TAMs (CD45^+^CD11b^+^F4/80^+^), M2-TAMs (CD45^+^CD11b^+^F4/80^+^CD206^+^), and MDSCs (CD45^+^CD11b^+^Gr-1^+^). **(B)** Gating strategies for T cells. **(C)** Gating strategies for Tregs (CD4^+^CD25^+^ Foxp3^+^).

Supplementary Figure 2 FMOs of some of the flow experiments **(A)** FMO of PD-1+T cells. **(B)** FMO of TIM-3+T cells. **(C)** FMO of PD-L1+ GL261 cells.
