# Supplementary figures and images for: Live-attenuated Japanese encephalitis virus inhibits glioblastoma growth and elicits potent antitumor immunity
Source: Front Immunol. 2023 Apr 11;14:982180. doi: 10.3389/fimmu.2023.982180 (PMC10126305; doi:10.3389/fimmu.2023.982180)

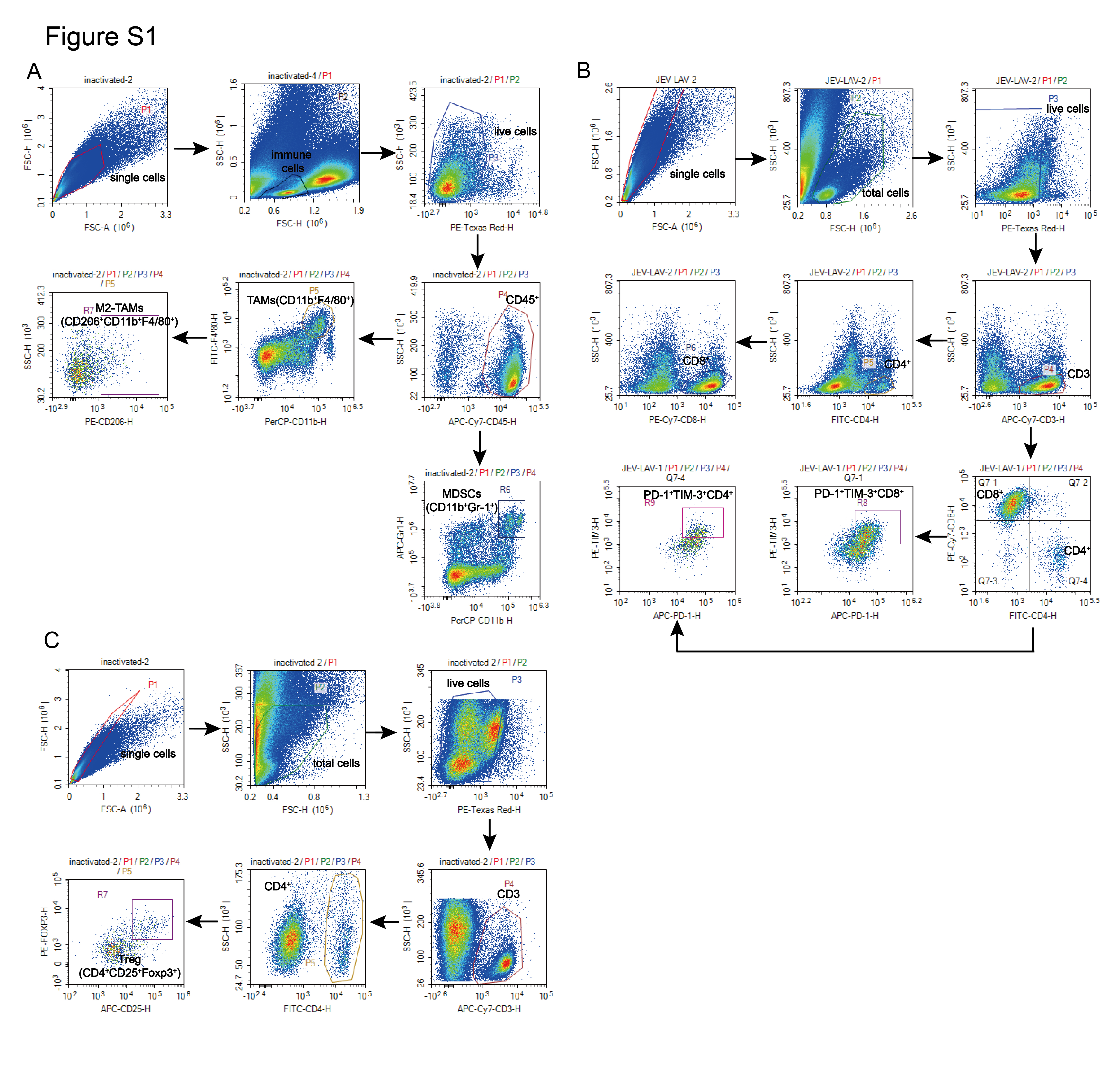

Supplement: Supplementary Figure 1 — Gating strategies for all flow experiments. (A) Gating strategies for CD45+cells, TAMs (CD45+CD11b+F4/80+), M2-TAMs (CD45+CD11b+F4/80+CD206+), and MDSCs (CD45+CD11b+Gr-1+). (B) Gating strategies for T cells. (C) Gating strategies for Tregs (CD4+CD25+ Foxp3+). [file Image_1.jpeg]

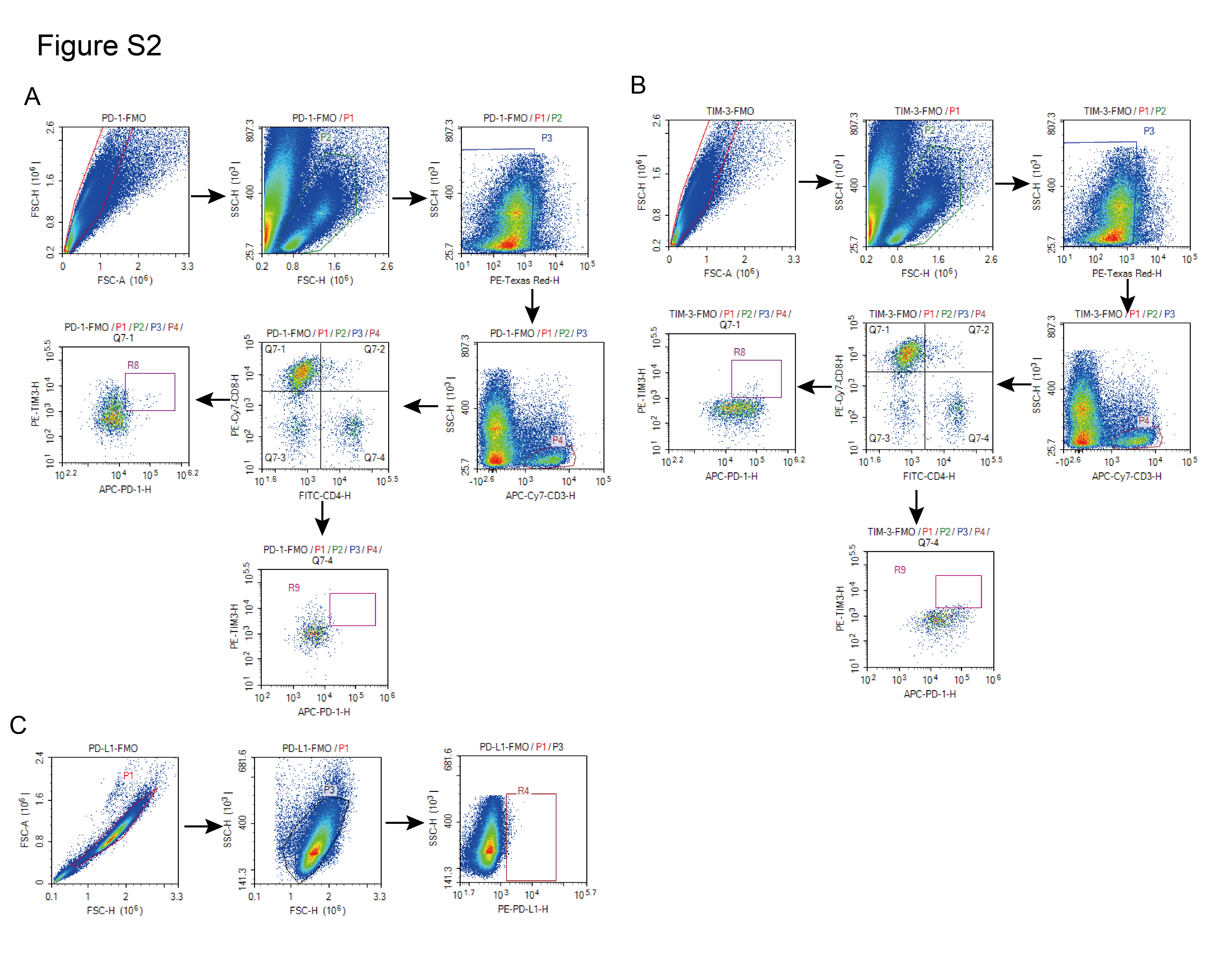

Supplement: Supplementary Figure 2 — FMOs of some of the flow experiments (A) FMO of PD-1+T cells. (B) FMO of TIM-3+T cells. (C) FMO of PD-L1+ GL261 cells. [file Image_2.jpeg]
